# Patient-related outcomes of patellofemoral arthroplasty: experience of a single center

**DOI:** 10.1186/s42836-021-00074-8

**Published:** 2021-06-03

**Authors:** W. Y. M. Abeysekera, W. Schenk

**Affiliations:** West Suffolk NHS Trust, Hardwick Ln, Bury St Edmunds, Bury Saint Edmunds, IP33 2QZ UK

**Keywords:** Patello-femoral arthroplasty, Patello-femoral joint arthritis, Knee joint arthritis, Knee joint arthroplasty

## Abstract

**Purpose:**

The purpose of this prospective study was to present the experience of a single center on patellofemoral arthroplasty, in terms of patient-related outcomes.

**Method:**

From January 2005 to January 2016, 42 patients with isolated patellofemoral osteoarthritis were treated. The patients were assessed using the Oxford Knee Score preoperatively, and one, five, and eight year(s) after surgery. The data of the patients were analyzed using linear mixed effects models. A *P* value of 0.05 was considered statistically significant.

**Results:**

Among 42 patients who underwent patellofemoral arthroplasty, only 25 patients (31 limbs involved) had records up to 5 years. There was a significant clinical improvement of Oxford Knee Score postoperatively (*P* < 0.05), lowering the score on average by 10.4 ± 1.5 one year after surgery and 8.9 ± 1.9 five years after surgery. This improvement was independent of the types of implants (*P* > 0.05), gender (*P* > 0.05), age (*P* < 0.05), and body mass index (BMI) (*P* < 0.05).

**Conclusion:**

Patellofemoral arthroplasty can significantly improve the knee function, and this improvement is independent of the type of implant, gender, age, and BMI. However, further studies will need to assess the long-term outcomes of PFA.

## Introduction

Osteoarthritis of the knee is a condition that causes significant disability. In the United Kingdom, over 300 million pounds is spent annually on knee arthroplasty. Isolated patellofemoral osteoarthritis (PFOA) is detected only 1 in 10 among the population with knee arthritis. These patients are usually relatively young [1].

In the early stages of the disease, there are non-operative treatments as for any other form of arthritis, but when it becomes severe, an operative approach might be an option. Arthroscopic debridement, patellectomy, total knee arthroplasty (TKA), and isolated patellofemoral arthroplasty (PFA) are the surgical alternatives for PFOA. Since only minimal benefit is attained in severe stages of the osteoarthritis, arthroscopic surgeries have become less popular, and patellectomy has resulted in poor long-term outcomes. As a result, TKA has become the optimal choice for isolated PFOA [2]. Such choice is further encouraged by the failure of the initial PFAs due to the residual patella malalignment, polyethylene wear, and failure secondary to disease progression of the rest of the knee joint [3]. However, the PFA has several advantages over the TKA for the isolated patellofemoral pathology. PFA allows a speedy recovery following the surgery, because it is a relatively less invasive procedure which preserves the natural ligaments, natural femur and tibia, and nearly normal function of the knee joint [4]. According to a randomized clinical trial evaluating 100 patients, the PFOA patients had a shorter recovery period, a greater range of motion, better physical function scores and less knee pain, compared to their TKA counterparts [5].

Recent studies have shown the enhanced outcomes due to better patient selection and more attention paid to soft tissue balancing. Recent developments in implant designs (second-generation PFA) and surgical techniques have also contributed to the significant improvement in short- and medium-term PFA results [6–12]. Athough the survival rates have improved dramatically with novel implants, according to several studies, some patients are still not satisfied with their experience [13].

In this prospective study, we presented the experience in PFA in a single center in terms of patient-related outcomes.

## Materials and methods

From January 2005 to January 2016, 42 consecutive patients with isolated PFOA were treated in our hospital. The inclusion criteria included isolated PFOA with severe discomfort in daily living, such as difficulty in moving up and down on stairs, sitting for a long time, rising from a low chair, and pain at rest. X-ray findings, specifically the level of femorotibial degenerative changes, were determined according to the radiological criteria described by Kellgren et al. [14]. The patients were assessed in terms of the Oxford Knee Score (OKS) preoperatively, and one, five, and eight year(s) after surgery.

All the patients received second-generation PFA implants (symmetric trochlear surface; Avon, Bristol, UK) or (asymmetric trochlear surface; Journey, Smith and Nephew, Memphis, Tennessee, USA). All operations were performed by three different consultant knee surgeons of the same department, but most were performed by the senior surgeon. All surgeons used both types of implants. It was up to the department to decide if to change from Avon to Journey implants, for several logistic and financial reasons.

The limited medial parapatellar approach was used in all cases. None of the surgeons employed the lateral para-patellar approach. Although patella resurfacing was not performed in all TKAs, the patella was resurfaced in all PFAs. We believe this make sense for the treatment of PFOA. In each surgery, apart from the basic arthroplasty principles, the surgeons strictly adhered to the surgical technique as described in the technical guides or instructions provided for the relevant implant. Particular care was taken not to overstuff the patellofemoral joint while achieving the proper patella tracking and patella stability by avoiding a valgus and/or internal rotation of implant components. In order to achieve this, a limited lateral release of the patella was performed in some cases based on the intraoperative assessment. Immediately after surgery, range of movement exercises (0° to 90°) were started as tolerated by the patient, and weight-bearing was restricted for 2 weeks.

In the follow-up study, we assessed surgery-related the general complications, such as urinary or respiratory tract infections, cardiovascular problems, and the complications specifically related to the PFA. The patients were asked to complete a self-reported OKS, which is a validated knee arthroplasty functional outcome measure consisting of 12 items related to the daily activities. The scores range from 12 to 60, with 12 representing the best outcome and 60 the worst outcome [15].

Statistical analysis package R (version 3.6.0) was used in this study. The data were recorded as mean ± standard deviation. Paired *t* tests were used to determine the mean OKS and differences, and the results were presented in bar charts. By using linear mixed effects models, the data of the patients with records up to 5 years (*n* = 25) were applied to estimate the overall OKS change across time and to test the significance of the factors such as the type of implant, age, sex, and body mass index (BMI). A *P* value of 0.05 was considered statistically significant.

## Results

During the given period, 42 patients underwent PFA. Only 33 patients (39 limbs) had responded to the review. Complete 5-year data were available from 25 patients (involving 31 limbs), and complete 8-year data were available in 6 patients (including 7 limbs). There were 14 left and 17 right knees among 27 females and 4 males. Sequential bilateral PFA were performed in 6 patients. Two types of implants were used, namely, Avon (*n* = 11) and Journey (*n* = 20). Only 2 complications were reported, one requiring a manipulation under anesthesia to overcome knee stiffness while the other needing a patella resurfacing after only the trochlea was resurfaced.

**Fig. 1:**
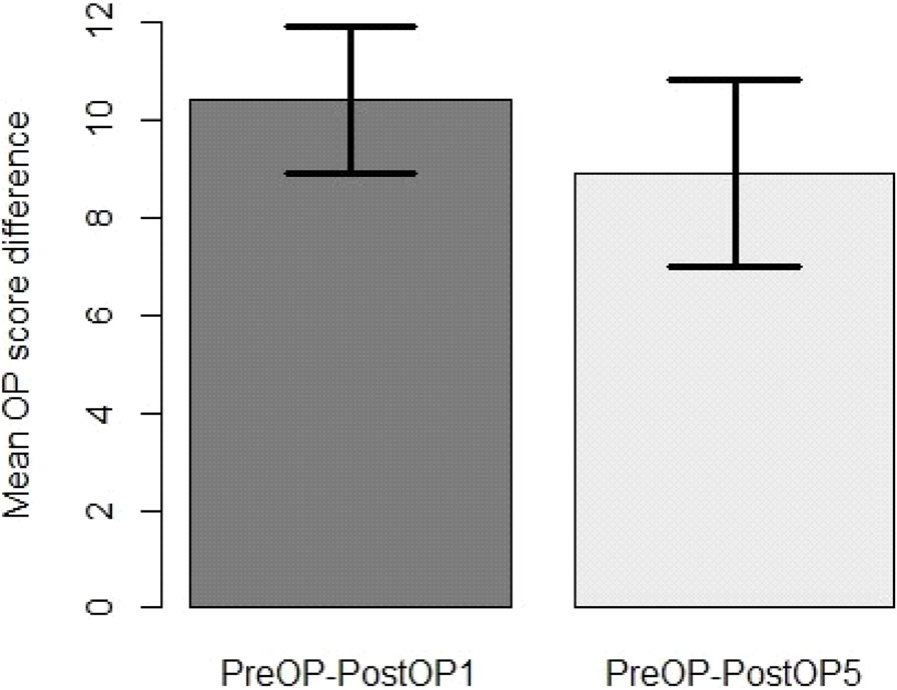
Mean OKS difference 1 and 5 year(s) after operation

**Fig. 2:**
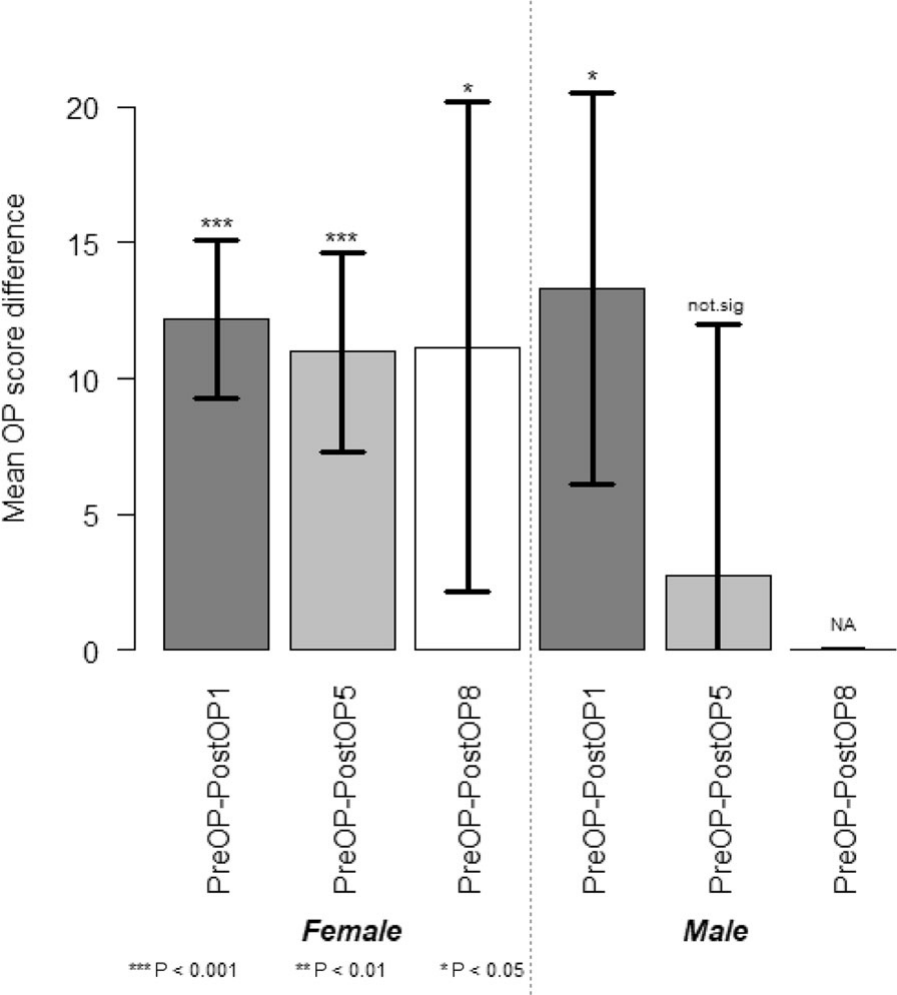
Mean OKS difference 1,5 and 8 year(s) after operation among males and females

**Fig. 3:**
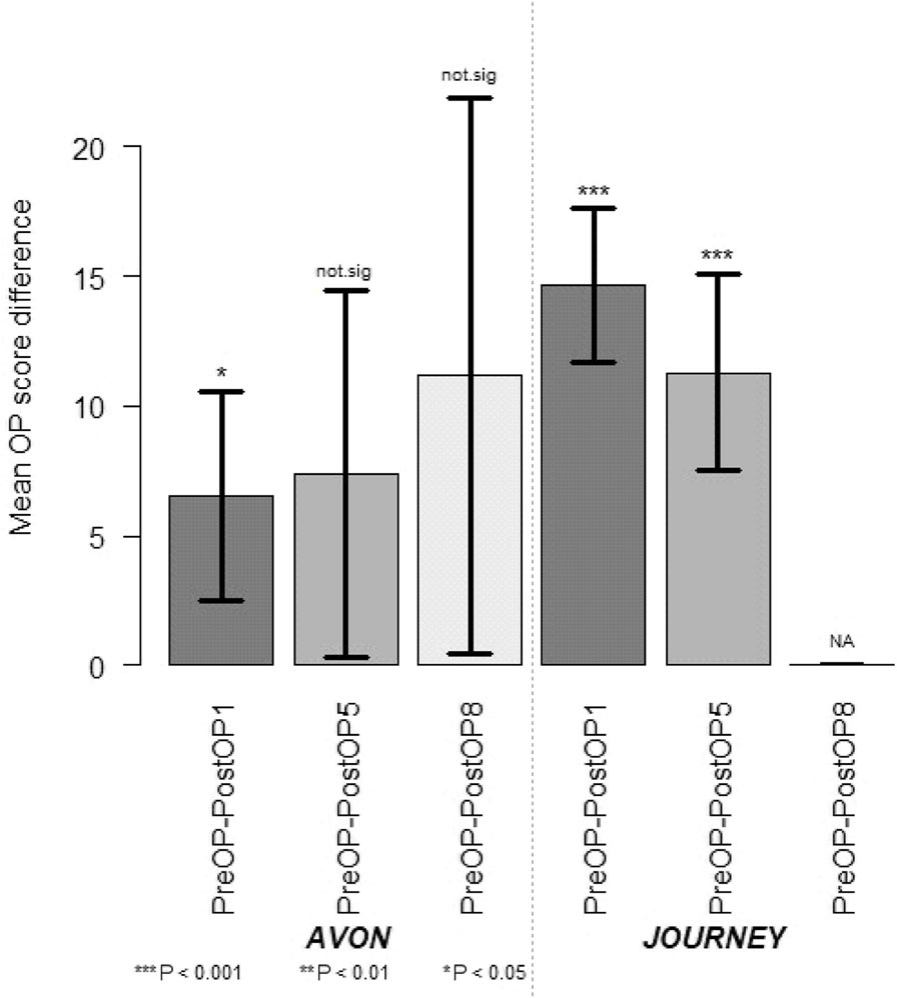
Mean OKS difference at 1,5 and 8 year(s) after operation  between two main different implants

Considering only patients (*n* = 25) who had records up to 5 years, the OKS significantly dropped over time (*P* = 9.996e-13 < < 0.05), lowering the score, on average, by 10.4 ± 1.5 one year after surgery and by 8.9 ± 1.9 five years after surgery, as estimated by using a linear mixed effects model (Fig. 1). Within each gender group, the OKS decreased after surgery, indicating a clinical improvement. It appeared that this improvement was more prominent in male patients than in female ones. However, the number of male patients was much smaller, and the analysis using the mixed effects models showed that there was no significant difference in the improvement pattern between the male and female patients (*P* = 0.78) (Fig. 2). The OKS significantly decreased with each type of implant after surgery. Although the mixed effects model analysis (*P* = 0.58) did not show a significant difference between the implants, improvement was more conspicuous with the Journey implants than with the Avon ones (Fig. 3).

**Fig. 4:**
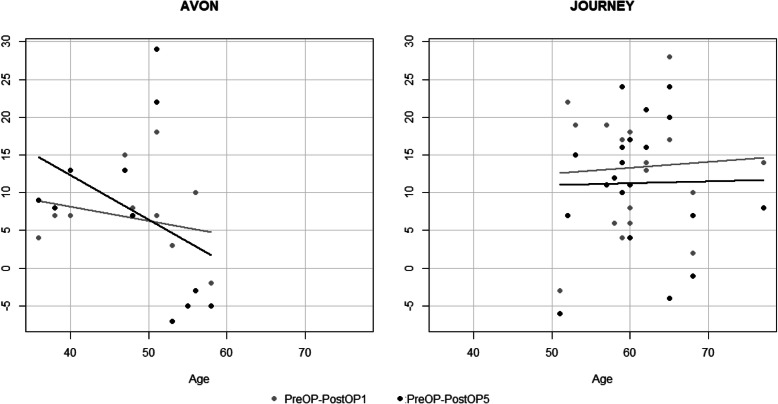
Postoperative OKS improvement in relation to the age among the two different implant types

**Fig. 5:**
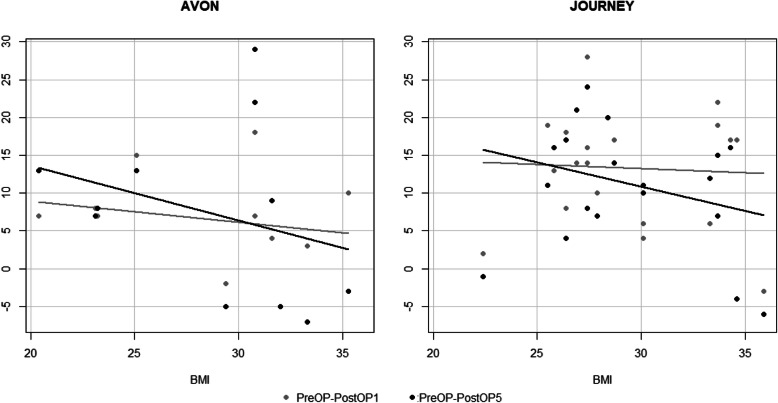
Postoperative OKS improvement in relation to the BMI among the two different implant types

The Avon implant was mostly used in younger patients, and the Journey implant was used in older patients. When the Avon implant was used, the improvement over time was less in older patients compared to younger patients. However, when the Journey implant was used, the improvement over time was more consistent and did not differ much in terms of the age of the patients. Generally, for both implants, the mixed effects analysis showed that improvement in OKS was not dependent on the age of patients (*P* = 0.85) (Fig. 4). In both types of implants, the improvement over time was more obvious in patients with lower BMI. Nonetheless, the mixed effects analysis showed that this improvement was no statistically significant (*P* = 0.11) (Fig. 5).

## Discussion

Some studies have shown inconsistent outcomes of PFA, including relatively high failure rates [6, 16–19]. However, Cartier et al. [20] reported an excellent functional outcome in 77% of patients after following up a retrospective case series for ten years. Similarly, Odumenya et al. [7] and Stark et al. [21] reported excellent functional outcomes with a 100% survival rate at 5 and 2 years of follow-ups, respectively. Those findings supported the value of PFA for patients with isolated PFOA. In a recently published systematic review, the overall survival rates of both first- and second-generation PFA implants were 92% at 5 years, 83% at 10 years, 75% at 15 years, and 67% at 20 years [22], respectively.

Our results showed a short-term improvement of knee function with the use of PFA in the isolated PFOA. It appeared that this improvement was more prominent in male patients than in females. There was a more evident improvement when the Journey implants were used as compared to the Avon. Most studies revealed that neither gender nor age influenced clinical or radiological outcomes [6, 23–25], which is similar to our findings. When the Avon implant was used, the improvement over time was less in older patients compared to younger patients. However, when the Journey implant was used, the improvement over time was more consistent and did not differ much in terms of the age of the patient.

Generally, it is assumed that obesity has a negative effect on the clinical outcomes of TKA [26, 27]. Similarly, a systematic review of 872 knees in 14 eligible studies [28] and another PFA study of 185 knees [6] highlighted a BMI > 30 kg/m^2^ as a patient characteristic relating to a poor outcome in PFA. Moreover, in a more recent retrospective study, Liow et al. [29] described a lower patient satisfaction with a slow improvement in functional outcomes in obese patients following PFA. Van Jonbergen et al. [25] reported a higher revision rate in obese patients. However, we did not find BMI affects the PFA outcomes.

Statistically, the sample size was too small to allow a comparison, which impairs the power of the study and increases the margin of error, which might render the study meaningless. Owing to the inherent features of the retrospective study, the postoperative assessments could not be blinded. In addition, surgeons’ preference, experience, and skills might affect the precise evaluation of the effects of the PFA.

## Conclusion

The PFA can significantly improve the knee function, and this improvement is independent of the type of implant, gender, age, and BMI. However further studies are warranted to assess the long-term outcomes of PFA.

## Data Availability

The datasets used and/or analyzed during the current study are available from the corresponding author on request.

## Abbreviations

PFA: Patello-femoral arthroplasty
PFJA: Patello-femoral joint arthritis
OKS: Oxford Knee Score
Post-OP: Postoperative
TKR: Total knee replacement
BMI: body mass index
